# The Dramatic Impact of Explicit Instruction on Learning to Read in a New Writing System

**DOI:** 10.1177/0956797620968790

**Published:** 2021-02-26

**Authors:** Kathleen Rastle, Clare Lally, Matthew H. Davis, J. S. H. Taylor

**Affiliations:** 1 Department of Psychology, Royal Holloway, University of London; 2 MRC Cognition and Brain Sciences Unit, University of Cambridge; 3 Division of Psychology and Language Sciences, University College London

**Keywords:** reading, learning, instruction, phonics, artificial language, open data, open materials

## Abstract

There is profound and long-standing debate over the role of explicit instruction in reading acquisition. In this research, we investigated the impact of teaching regularities in the writing system explicitly rather than relying on learners to discover these regularities through text experience alone. Over 10 days, 48 adults learned to read novel words printed in two artificial writing systems. One group learned spelling-to-sound and spelling-to-meaning regularities solely through experience with the novel words, whereas the other group received a brief session of explicit instruction on these regularities before training commenced. Results showed that virtually all participants who received instruction performed at ceiling on tests that probed generalization of underlying regularities. In contrast, despite up to 18 hr of training on the novel words, less than 25% of discovery learners performed on par with those who received instruction. These findings illustrate the dramatic impact of teaching method on outcomes during reading acquisition.

Reading acquisition involves learning individual printed words as well as the underlying regularities of a writing system. These regularities are characterizations of how the visual symbols of writing map onto the sounds and meanings of spoken language. One important regularity in alphabetic writing systems is the relationship between spellings and sounds (e.g., the letter “B” maps onto the sound /b/). There has been substantial interest in how we acquire this spelling-to-sound mapping (e.g., Seymour, Aro, Erskine, & COST Action A8 Network, 2003), but there are also powerful regularities between spelling and meaning conveyed through morphology (e.g., “-ed” at the end of a word indicates the past; Ulicheva, Harvey, Aronoff, & Rastle, 2020). Literate individuals use knowledge of both spelling-to-sound and spelling-to-meaning regularities to *generalize*, that is, to interpret words that they have not seen before, such as when pronouncing a nonword (e.g., “chilb”) or understanding an unfamiliar word (e.g., “TikTokker”).

There is wide agreement that learning these regularities is vital in reading acquisition, but there is also profound and long-standing debate about how this learning should be supported (e.g., Castles, Rastle, & Nation, 2018). One approach advocates explicit instruction of the regularities. For example, in systematic phonics training, a child may be taught that “D” makes the sound /d/ and then asked to read aloud examples such as “dog,” “dad,” and “dig.” An alternative approach suggests that these regularities may be discovered without explicit instruction, simply through text experience. Discovery learning is an important concept within constructivism (e.g., Bruner, 1961), in which it is argued that “knowledge [that] students construct on their own . . . is more valuable than the knowledge that is modeled for them; told to them; or shown, demonstrated, or explained to them” (Loveless, 1998, pp. 285–286). This view is frequently articulated in the popular media; for example, Michael Rosen, former Children’s Laureate of the United Kingdom, has argued that children discover regular patterns for themselves through experience and may therefore not require explicit instruction:

We sit with our children reading whole books, talking about them, sometimes pointing at whole words, sometimes at letters. We sit with them writing shopping lists, labelling things in their rooms, doing texting on phones, planning holidays looking at pictures and reading out the names of places . . . these are ways in which many people . . . have learned in part or whole how to read. (Rosen, 2013, para. 19)

The notion that regular patterns may be discovered through text experience appears consistent with a vast body of research on statistical learning. One of the most important findings of the past 20 years of psychological research is that infants (e.g., Saffran, Aslin, & Newport, 1996) and adults (e.g., Siegelman & Frost, 2015) extract information about regularities in the environment. These demonstrations of statistical learning tend to be rather circumscribed (Frost, Armstrong, & Christiansen, 2019), although there is evidence that similar learning processes arise in reading acquisition. Training studies demonstrate that children (Apfelbaum, Hazeltine, & McMurray, 2013) and adults (Taylor, Davis, & Rastle, 2017) can discover simple underlying regularities through experience with whole words and use this knowledge to generalize. Similarly, studies of generalization in children (Treiman & Kessler, 2019) and adults (Ulicheva et al., 2020) reveal knowledge of statistical regularities in the writing system that are not often taught explicitly, such as context-sensitive spelling-to-sound regularities (e.g., the pronunciation of initial “G” is influenced by the following vowel) and morphological regularities (e.g., word-final “-ous” conveys adjective status). Finally, the most sophisticated models of reading acquisition learn underlying regularities through experience with whole words (e.g., Harm & Seidenberg, 2004).

Remarkably little is known about how explicit instruction influences the learning of underlying regularities beyond these basic statistical-learning processes. Several artificial-language-learning studies have shown benefits of explicit instruction in acquiring knowledge of underlying patterns. However, design issues limit the conclusions that can be drawn. Many of these studies included few trained items (between 4 and 18) paired with features that may hinder discovery processes, such as use of visual symbols in as few as one word (Bishop, 1964; Jeffrey & Samuels, 1967) or inclusion of highly complex mappings in which two or three symbols were combined in different orders to represent single phonemes within a word (Bitan & Booth, 2012; Bitan & Karni, 2003). Other studies used training and testing protocols that do not resemble reading experience, such as short periods of passive exposure (Yoncheva, Blau, Maurer, & McCandliss, 2010) or use of forced-choice tests to probe generalization (Bitan & Booth, 2012; Bitan & Karni, 2003). The instruction manipulation in another study was confounded with the nature of the writing system; instructed participants learned a systematic writing system, but discovery-learning participants learned an arbitrary writing system (Yoncheva, Wise, & McCandliss, 2015). Finally, several of these studies had low participant numbers (e.g., 9; Bitan & Karni, 2003), and none accounted for item-based variation in the analysis of behavioral data.

**Statement of Relevance**

Learning to read is the most important milestone of a child’s education, but there is profound debate about how reading should be taught. We report a laboratory simulation of reading acquisition in which adults, over a period of 10 days, learned to read new words printed in unfamiliar writing systems. One group received explicit instruction about the structure of the writing systems before training, and one group discovered this structure through their reading experience. Both groups learned the trained words to a high standard over approximately 18 hr of training. However, instruction had a dramatic impact on participants’ ability to read and understand new, untrained words; less than 25% of discovery learners achieved the same standard as those who had received explicit instruction. These findings have important implications for literacy policy and practice because they suggest that providing explicit instruction may be transformative in helping all learners to become skilled readers.

The present study simulated the impact of explicit instruction on learning to read. We trained two groups of adults to read novel words in two artificial alphabets comprising different underlying spelling–sound and spelling–meaning regularities. Both groups received 10 days of training on learning to read 48 novel words, but for one group, the initial day of orthography training was substituted for a short session of explicit instruction on the structure of the two writing systems. Behavioral tests administered at the end of training assessed how this short period of explicit instruction influenced participants’ learning of underlying regularities, as well as their retention of the individual trained items.

## Method

### Data availability

Stimulus materials, instructions, trial-level data, and analysis scripts are available on OSF (https://osf.io/rtx5j/).

### Design

The study had a 2 × 2 design manipulating two factors: group and semantic marker. The group factor was manipulated between participants and referred to the nature of instruction received; one group received explicit instruction on the underlying regularities of the writing system (*explicit-instruction group*), whereas the other group discovered these regularities through experience with whole words (*discovery-learning group*). The semantic-marker factor was manipulated within participants and referred to the nature of the artificial alphabet learned. Both participant groups learned to read novel words printed in two artificial alphabets. Novel words were always composed of four symbols, and the final symbol was silent. In one of the alphabets, the final symbol of each word referred to its semantic category (*systematic language*); in the other alphabet, the final symbol of each word was meaningless (*arbitrary language*). The other symbols within both alphabets mapped consistently to the sounds within each novel word.

### Participants

Participants were 50 native-English speakers (39 women) between the ages of 18 and 35 years from Royal Holloway, University of London. Participants reported no history of language or literacy impairment and were paid for their time. Twenty-four participants were assigned to the discovery-learning condition, and 26 were assigned to the explicit-instruction condition. One participant from the explicit-instruction condition dropped out of the study following pretraining, and another from this condition was excluded from all analyses because of noncompliance (i.e., chance-level performance on button-press tasks coupled with very rapid responding). Participants in the discovery-learning condition were involved in 2 days of MRI scanning following training; data from those MRI scans are reported by Taylor, Davis, and Rastle (2019). Participant numbers were based on our previous artificial-language-learning research (*N* = 24; Taylor et al., 2017), in which participants showed good item-based learning following 10 days of training on 48 novel words printed in two artificial alphabets. Though we did not have a reliable estimate of the size of the between-groups instruction manipulation, we have shown in previous artificial-language-learning studies using these same participant numbers that between-groups effects can be observed (Lally, Taylor, Lee, & Rastle, 2019).

### Materials

**Fig. 1.**
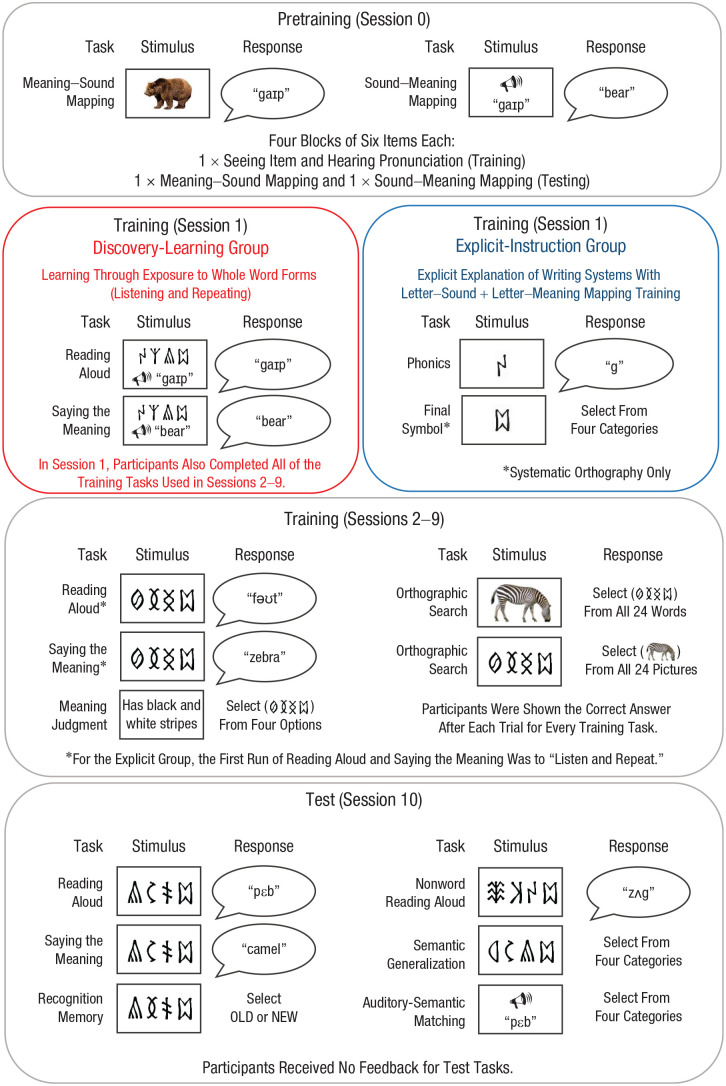
Training and testing protocol. The training tasks were constructed to build knowledge of individual novel words and were accompanied by feedback on every trial. The test tasks probed both knowledge of the trained novel words (reading aloud, saying the meaning, recognition memory, auditory-semantic matching) and knowledge of underlying regularities used to generalize (nonword reading aloud, semantic generalization).

Twenty-four novel words were designed in each of two artificial alphabets. These novel words were associated with phonological and semantic forms (see Fig. 1 for examples).

#### Phonological forms

Twenty-four consonant-vowel-consonant phonological forms were constructed for each of two languages. They were built from eight consonants (/b/, /f/, /g/, /m/, /p/, /t/, /v/, /z/) and eight vowels, four of which were used in each language (/ԑ/, /Ʌ/, /ai/, /ƏƱ/ and /æ/, /ɒ/, /i/, /u/). Consonants occurred three times in onset and coda positions for each language, and vowels occurred six times each. Stimuli were recorded by a female native-English speaker at a sampling rate of 44.1 kHz.

#### Orthographic forms

Twenty visual symbols were selected from each of two archaic scripts (Hungarian runes and Georgian Mkhedruli). Sixteen of the symbols from each script were associated with the 16 phonemes comprising the novel words. The other four symbols for each script were silent, and each occurred in the final position of six novel words. Orthographic forms of each trained word were constructed using the symbols from both scripts, and the assignment of script to language was counterbalanced across participants.

#### Semantic forms

Two sets of 24 color pictures depicting familiar objects were selected. Each set comprised six exemplars in each of four categories: animal, fruit or vegetable, vehicle, and tool. For the systematic language, each of the four final silent symbols was associated with one semantic category. For the arbitrary language, the assignment of final symbol to semantic category was random. Semantic markers (usually termed *semantic classifiers*) are frequently encountered in East Asian languages (e.g., Bengali, Burmese, Chinese, Japanese, Thai, Vietnamese) and can denote properties such as number, animacy, shape, function, and orientation. The assignment of object set to language and the assignment of orthography to the systematic/arbitrary distinction was counterbalanced across participants.

#### Test-task items

Three additional sets of 24 items were constructed in each language for the test tasks. The first set (recognition-memory phonological distractors) changed only the vowel, the second set (recognition-memory semantic distractors) changed only the final symbol, and the third set (nonword reading aloud and semantic generalization) changed both the vowel and final symbol.

### Procedure

The training and testing protocol is displayed in Figure 1. Participants completed 11 consecutive days of behavioral training and testing (hereafter, referred to as *sessions*) with breaks for weekends. Session 0 involved spoken-language training, Sessions 1 through 9 involved orthography training, and Session 10 involved testing. The training tasks were designed to build participants’ knowledge of the novel words. The test tasks were designed to probe both knowledge of the novel words and knowledge of the underlying regularities in the writing systems. Stimulus presentation and data recording were controlled by E-Prime software (Version 2.0; Schneider, Eschman, & Zuccolotto, 2012). Choice responses were collected via key press or mouse click, whereas spoken responses were recorded and manually coded for accuracy off-line.

#### Spoken-language training (Session 0)

Participants learned sound–meaning associations for the novel words in each language. The words in each language were divided into four blocks of six words, and participants completed three runs of each block. Blocks consisted of training trials, in which participants saw a picture of each item while hearing its pronunciation, followed by two types of testing trial, in which they verbally produced the spoken form for a picture and verbally produced the English meaning for a spoken form.

#### Instruction (Session 1)

Instead of orthography training, the explicit-instruction group participated in a short instruction session. They first received a PowerPoint presentation showing how the visual symbols in the two languages mapped to sounds and meanings. They then received four runs of phonics training. In the first run, each visual symbol was displayed with its sound. In the subsequent three runs, participants were prompted to produce the sound in response to each visual symbol; they then heard the correct sound as feedback. Finally, for the systematic language, they completed one run of symbol-to-picture matching and one run of picture-to-symbol matching (both with feedback). The experiment was otherwise identical for the two groups.

#### Orthography training (Sessions 1–9 or 2–9)

Orthography training was provided for approximately 90 to 120 min per day and comprised four tasks. Orthographies were presented in alternating blocks during the training tasks, and the order of tasks varied across days. Participants received the correct answer on every trial for every task following their response.

The first training task was *reading aloud* (24 trials, four repetitions per orthography). Participants were shown each printed word in a randomized order and asked to read it aloud. In Session 1 for the discovery-learning group and Session 2 for the explicit-instruction group, the first block involved seeing the written form and hearing and repeating the spoken form. The second task was *saying the meaning* (24 trials, four repetitions per orthography). Participants were shown each printed word and asked to say its meaning. In Session 1 for the discovery-learning group and Session 2 for the explicit-instruction group, the first block involved seeing the written form and hearing and repeating the meaning. The third task was *orthographic search* (48 trials per orthography). Participants saw a picture and then selected the orthographic form from a grid of 24 items; they then completed the task in reverse. The final training task was *meaning judgment* (72 trials per orthography). Participants read a sentence describing the appearance, function, location, or taste of an item and selected the appropriate trained word from a selection of four orthographic forms. Orthographic forms included the target, a same-category distractor, and two distractors from another semantic category.

#### Testing (Session 10)

Participants completed six test tasks for each language. Each trained item was presented only once in each task (the exception was recognition memory, in which trained items were presented twice to yield equal numbers of “yes” and “no” responses), and no feedback was provided. Items within test tasks were presented in randomized order for each participant, and languages were presented in separate blocks for all test tasks.

The first test task was *reading aloud*, in which participants read aloud each of the 24 trained novel words for each language, and the second was *saying the meaning*, in which participants produced the English meaning of each of the 24 trained novel words for each language. In the third test task, *nonword reading aloud*, participants read aloud 24 untrained novel words for each language. This task assessed participants’ knowledge of underlying spelling–sound regularities. The fourth test task was *recognition memory*, in which participants decided whether or not they had previously studied visually presented items. The fifth was *auditory-semantic matching*, in which participants heard the spoken form of each trained novel word and decided to which of four semantic categories it belonged. In the sixth task, *semantic generalization*, participants saw 24 untrained novel words from each language and decided to which of four semantic categories they belonged. This task probed participants’ knowledge of underlying spelling–meaning regularities. Participants completed this task for both languages, although there was no correct answer for the arbitrary language.

## Results

Training and test data were analyzed using the *lme4* package for generalized mixed models (Version 1.1-20; Bates, Mächler, Bolker, & Walker, 2015) in the R programming environment (Version 3.5.2; R Core Team, 2016). Models were specified in a hypothesis-driven manner by including fixed factors of interest for each training and test task. These typically included group (discovery learning vs. explicit instruction), semantic marker (systematic vs. arbitrary), and session. Random factors for participants were included in each analysis. The extensive counterbalancing of stimuli meant that there was no single factor able to capture item-based variation. Instead, we included spoken word, script, and meaning as random factors, as appropriate. βs and odds ratios (*OR*s) are used to report effect sizes. β is the logit-transformed fixed-effect coefficient, which refers to the estimated difference between conditions after analyses controlled for random effects. *OR* (derived from β) measures the difference in odds of being correct (vs. incorrect) in one level of a fixed effect compared with another. For fixed effects of group, *OR* compares the odds of being correct (vs. incorrect) in the explicit-instruction group relative to the discovery-learning group (e.g., *OR* = 5.0 would mean that the odds of being correct are five times greater for the explicit-instruction group relative to the discovery-learning group; Osborne, 2008). The same principle applies when comparing items with systematic markers relative to arbitrary markers. For fixed effects of session, *OR* denotes the change in odds for participants being correct (vs. incorrect) for every additional training session.

In the following section, we summarize the results of analyses relating to our training and test tasks and supplement these with figures displaying average performance and variability. Readers interested in further details about model construction and the random-effects structure may access data, analysis scripts, and full output of the models on OSF (https://osf.io/rtx5j/).

### Spoken-language training (Session 0)

Analyses treated group as a fixed factor and participant, spoken word, and meaning as random factors. Results showed that participants in the discovery-learning and explicit-instruction groups learned the spoken words to a moderate degree of accuracy; there were no significant differences between groups either in the saying-the-meaning component (proportion correct = .63 vs .64, respectively; β = 0.12, *OR* = 1.13, *SE* = 0.32, *Z* = 0.38, *p* = .704) or in the saying-the-spoken-form component (proportion correct = .62 vs .67, respectively; β = 0.38, *OR* = 1.46, *SE* = 0.31, *Z* = 1.22, *p* = .222) of the task.

### Orthography training (Sessions 1–9)

**Fig. 2.**
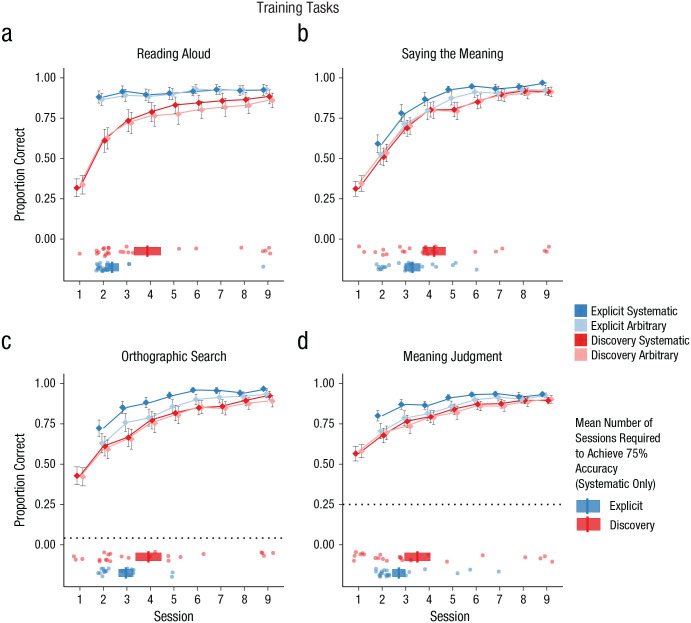
Performance on the four training tasks: (a) reading aloud, (b) saying the meaning, (c) orthographic search, and (d) meaning judgment. The proportion of correct responses is shown for each task as a function of session, participant group (explicit instruction vs. discovery learning), and the nature of the semantic marker (systematic vs. arbitrary). Dotted lines show chance performance on the orthographic-search and meaning-judgment tasks. Error bars display ±1 *SE*, calculated for between-subjects designs in order to compare group performance. The horizontal boxplots in the lower half of each graph show the mean number of sessions required for participants to achieve 75% accuracy in the systematic language (vertical bars). The left and right edges of each box indicate ±1 *SE*, calculated for between-subjects designs in order to compare group performance. Data points indicate the number of sessions each individual needed to achieve 75% accuracy. Note that the explicit-instruction group did not complete any of the training tasks in Session 1.

Results for orthography-training tasks are displayed in Figure 2. Analyses of all training tasks included group, semantic marker, and session as fixed factors and participant, spoken word, meaning, and script as random factors. Note that the explicit-instruction group did not participate in the orthography training in Session 1.

#### Reading aloud

Performance on the reading-aloud training task is shown in Figure 2a. Participants in the explicit-instruction group showed superior reading-aloud accuracy (β = 1.39, *OR* = 4.01, *SE* = 0.44, *Z* = 3.14, *p* = .002). Performance improved across sessions (β = 0.46, *OR* = 1.58, *SE* = 0.01, *Z* = 62.04, *p* < .001), although this improvement was more pronounced in the discovery-learning group (Session × Group interaction, β = −0.34, *OR* = 0.71, *SE* = 0.01, *Z* = −27.87, *p* < .001) because performance of the explicit-instruction group was near ceiling from the beginning of training. Finally, there was a significant effect of semantic marker (β = 0.18, *OR* = 1.20, *SE* = 0.03, *Z* = 5.95, *p* < .001), although the systematicity advantage was greater in the discovery-learning group (Semantic Marker × Group interaction, β = −0.28, *OR* = 0.75, *SE* = 0.05, *Z* = −5.18, *p* < .001). This may indicate that although the explicit-instruction group was aware that the final symbol was silent for both languages, the discovery-learning group was more likely to discover that the final symbol was silent in the systematic language than in the arbitrary language, and this information supported their reading-aloud performance.

#### Saying the meaning

Performance on the saying-the-meaning training task is shown in Figure 2b. Performance improved across sessions (β = 0.54, *OR* = 1.72, *SE* = 0.01, *Z* = 73.20, *p* < .001). There was also evidence that participants in the explicit-instruction group were able to use knowledge of the final symbol to improve their performance (Semantic Marker × Group interaction, β = 0.60, *OR* = 1.82, *SE* = 0.05, *Z* = 12.24, *p* < .001). This contrasts with the reading-aloud results; in this task the explicit-instruction group used information about the semantic marker to improve performance for the systematic language because the instruction indicated that the final symbol was relevant to meaning for that language. There were no other significant effects or interactions.

#### Orthographic search

Performance on the orthographic-search training task is shown in Figure 2c. Performance was collapsed for simplicity across the two components of the orthographic-search task (selecting a picture and selecting the orthographic form), so trials from both of these components were included in the same model. Performance improved across sessions (β = 0.46, *OR* = 1.59, *SE* = 0.01, *Z* = 47.83, *p* < .001). There was also evidence that performance was superior when there was a systematic semantic marker (β = 0.11, *OR* = 1.11, *SE* = 0.04, *Z* = 2.61, *p* = .009), although this effect was more pronounced in the explicit-instruction group (Semantic Marker × Group interaction, β = 0.61, *OR* = 1.84, *SE* = 0.07, *Z* = 8.96, *p* < .001). Once again, the explicit-instruction group used information about the final symbol in this task because they knew it was relevant to meaning. There were no other significant effects or interactions.

#### Meaning judgment

Performance on the meaning-judgment training task is shown in Figure 2d. Performance improved across sessions (β = 0.29, *OR* = 1.34, *SE* = 0.01, *Z* = 42.96, *p* < .001), although improvement was steeper in the discovery-learning group (Group × Session interaction, β = −0.04, *OR* = 0.96, *SE* = 0.01, *Z* = −3.38, *p* < .001) because the explicit-instruction group was more accurate from the beginning of training. As in the other meaning-based tasks, there was evidence that participants in the explicit-instruction group used their knowledge of the final symbol to improve performance (Semantic Marker × Group interaction, β = 0.40, *OR* = 1.49, *SE* = 0.05, *Z* = 7.64, *p* < .001). There were no other significant effects or interactions.

#### Instructional time

The data in Figure 2 suggest that participants in both groups learned the trained items to a high degree of accuracy by Session 9 and that performance on some tasks was superior earlier in training for the explicit-instruction group. However, it is also possible to interpret the training data in a different manner, by asking how many sessions it took for participants to reach a good standard of accuracy and whether this was influenced by instruction. The horizontal boxplots in Figure 2 display the mean session number at which accuracy reached 75% for each instruction group, and the data points around the boxplots refer to individual performance. Session 1 was the first day of orthography training for the discovery-learning group; Session 2 was the first day of orthography training for the explicit-instruction group (these participants had received instruction in lieu of orthography training in Session 1). We considered only the systematic language, as this is the language in which the impact of instruction should be greatest.

The explicit-instruction group required fewer sessions on average to reach 75% correct performance on all four tasks. However, when data were analyzed using between-subjects *t* tests, this difference in instruction time reached statistical significance only in the reading-aloud task, *t*(46) = −2.34, *p* = .024, *d* = 0.68. Differences were not statistically significant in the saying-the-meaning task, *t*(46) = −1.53, *p* = .132, *d* = 0.44; orthographic-search task, *t*(46) = −1.51, *p* = .138, *d* = 0.44; or meaning-judgment task, *t*(46) = −1.27, *p* = .211, *d* = 0.37. It may be that explicit instruction on regular patterns in the writing system impacts the speed of learning new words when the task can be achieved through knowledge of those regular patterns (as is the case for reading aloud). Explicit instruction provided no significant reduction in instruction time for those tasks that required at least some degree of specific item-based knowledge.

### Testing (Session 10)

**Fig. 3.**
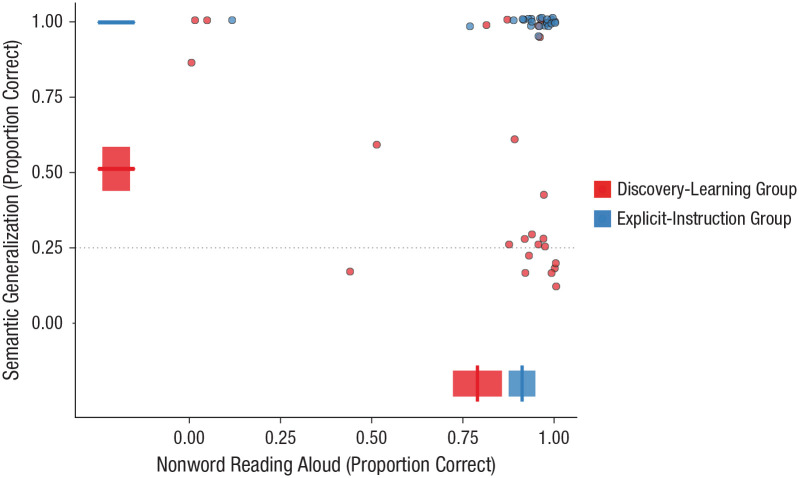
Relationship between performance on the nonword-reading-aloud and semantic-generalization tasks at the group and participant levels, separately for the discovery-learning and explicit-instruction groups. Data points display generalization accuracy for individual participants. Boxplots display group-level performance on nonword reading aloud (averaged across systematic and arbitrary languages) and semantic generalization (systematic language only). Each boxplot shows the mean (vertical bar on the *x*-axis or horizontal bar on the *y*-axis) and ±1 *SE* (left and right edges of the box on the *x*-axis or upper and lower edges on the *y*-axis), calculated for between-subjects designs in order to compare group performance.

Performance on the nonword-reading-aloud task and semantic-generalization task is shown in Figure 3. These test tasks provide an index of participants’ learning of spelling–sound and spelling–meaning regularities within the writing systems. Figure 3 displays group-level performance and performance of individual participants averaged across systematic and arbitrary languages in the nonword-reading-aloud task and for the systematic language only in the semantic-generalization task (there was no correct answer for the arbitrary language in this task).

#### Nonword reading aloud

Performance on the nonword-reading-aloud test task is shown along the *x*-axis of Figure 3 (averaged across systematic and arbitrary languages). Performance is broken down further into systematic and arbitrary languages in Figure S1 in the Supplemental Material available online. Analyses included group and semantic marker as fixed factors and participant, spoken word, and script as random factors. Critically, performance was superior in the explicit-instruction group (β = 1.45, *OR* = 4.24, *SE* = 0.73, *Z* = 1.99, *p* = .047). The *OR* for this result indicates that the odds of being correct (vs. incorrect) were more than four times greater in the explicit-instruction group relative to the discovery-learning group. There was also a positive influence of the systematic semantic marker on reading-aloud performance (β = 0.47, *OR* = 1.60, *SE* = 0.22, *Z* = 2.13, *p* = .033; see Fig. S1). Though there was no interaction with group, this effect of semantic marker appears to be driven by performance in the discovery-learning group. In accordance with our interpretation of the results in the reading-aloud training task (Fig. 2a), we believe that this suggests that participants in the discovery-learning group were more likely to learn that the final symbol was silent in the systematic language than in the arbitrary language, and they used this information to improve performance on the nonword-reading-aloud task. There were no other significant effects or interactions.

#### Semantic generalization

Performance on the semantic-generalization test task is shown along the *y*-axis of Figure 3 (systematic language only; there was no correct answer for the arbitrary language in this task). Analyses on the systematic language included group as a fixed factor and participant and spoken word as random factors. Critically, performance was again superior in the explicit-instruction group (β = 7.77, *OR* = 2,374.25, *SE* = 1.52, *Z* = 5.12, *p* < .001). The *OR* for this result indicates that the odds of being correct (vs. incorrect) were many times greater in the explicit-instruction group relative to the discovery-learning group. Data plotted in Figure 3 indicate that nearly half of the discovery-learning participants performed at chance in this task, but performance was at ceiling for all participants in the explicit-instruction group.

Data provided in Figure 3 reveal that nearly all participants in the explicit-instruction group performed at ceiling on both tasks, indicating that they had learned the underlying spelling–sound and spelling–meaning regularities of the writing system. Performance of the discovery-learning group was clearly very different. Despite having up to 18 hr of orthography training on the 48 novel words over a 2-week period, very few of the discovery-learning participants uncovered both forms of regularity to a sufficient degree to generalize (indicated by performance in the upper right corner of the plot). It is also striking that performance in this group did not fall along the diagonal of the plot, which would have indicated learning of both regularities to at least some degree. Instead, most participants in the discovery-learning group uncovered one regularity but not the other (indicated by performance in the bottom right and top left corners of the plot).

**Fig. 4.**
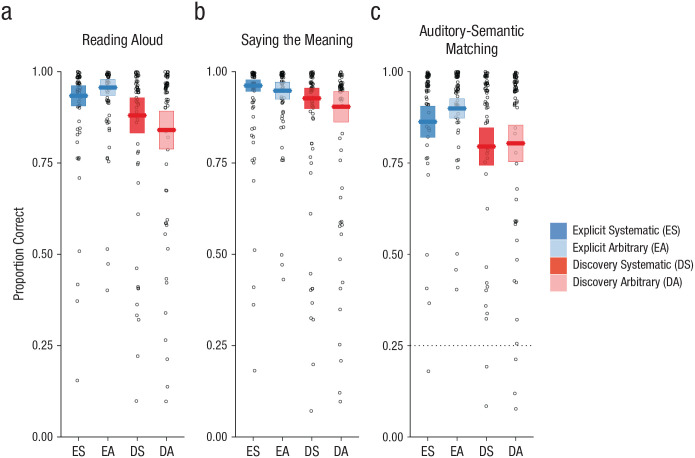
Performance on the three test tasks: (a) reading aloud, (b) saying the meaning, and (c) auditory-semantic matching. The proportion of correct responses is shown for each task as a function of participant group (explicit instruction vs. discovery learning) and nature of the final symbol (systematic vs. arbitrary). The dotted line shows chance performance on the auditory-semantic matching task. Each boxplot displays the mean (horizontal bar) and ±1 *SE* (upper and lower edges of the box), calculated for between-subjects designs in order to compare group performance. Data points display mean performance for individual participants.

The very stark differences between the discovery-learning and explicit-instruction groups in generalization performance may reflect poor learning of individual items in the discovery-learning group. However, this explanation is inconsistent with analyses of test tasks conducted in Session 10 that assessed learning of the trained items. These analyses showed a high degree of accuracy on trained items for both groups and null or moderate effects of instruction condition. Data from these test tasks are visualized in Figure 4.

#### Reading aloud

Performance on the reading-aloud test task is shown in Figure 4a. Participants in both groups showed a high degree of accuracy in reading aloud trained novel words, although performance in the explicit-instruction group was superior (β = 2.11, *OR* = 8.25, *SE* = 0.74, *Z* = 2.83, *p* = .005). There was some evidence for an effect of semantic marker (β = 0.54, *OR* = 1.72, *SE* = 0.23, *Z* = 2.33, *p* = .020) that was larger in the discovery-learning group (Semantic Marker × Group interaction, β = −1.69, *OR* = 0.19, *SE* = 0.44, *Z* = −3.86, *p* < .001). Model comparisons within each group revealed a significant effect of semantic marker in the discovery-learning group (β = 0.54, *OR* = 1.72, *SE* = 0.22, *Z* = 2.42, *p* = .016), but not the explicit-instruction group (β = −0.62, *OR* = 0.54, *SE* = 0.33, *Z* = −1.91, *p* = .056), mirroring the training data.

#### Saying the meaning

Performance on the saying-the-meaning test task is shown in Figure 4b. Participants in both groups showed a high degree of accuracy in retrieving the meanings of visually presented trained novel words. There were no significant effects or interactions in this task.

#### Auditory-semantic matching

Performance on the auditory-semantic matching test task is shown in Figure 4c. Participants in both groups showed a high degree of accuracy in verifying the meanings of aurally presented trained novel words. There were no significant effects or interactions in this task.

#### Recognition memory

**Fig. 5.**
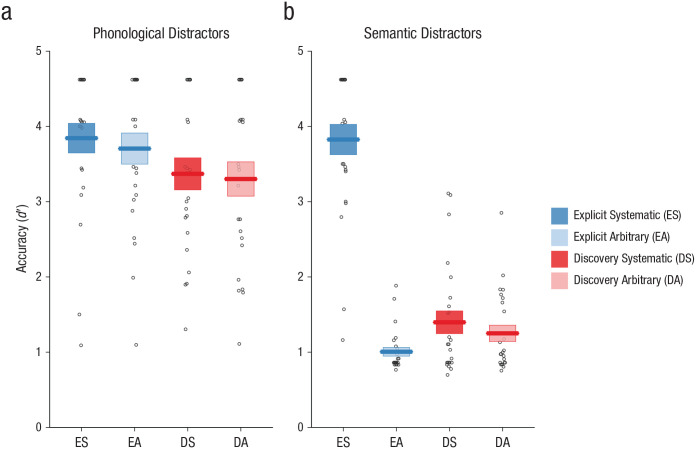
Performance on the recognition-memory task as a function of participant group (explicit instruction vs. discovery learning) and nature of the semantic marker (systematic vs. arbitrary), separately for (a) phonological distractors and (b) semantic distractors. Each boxplot displays the mean (horizontal bar) and ±1 *SE* (upper and lower edges of the box), calculated for between-subjects designs in order to compare group performance. Data points display mean performance for individual participants. Performance is described using the *d*′ sensitivity index: *d*′ = *z*(hit rate) – *z*(false-alarm rate). Phonological distractors were the same as trained items apart from the vowel (e.g., 
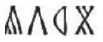
 rather than 
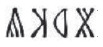
). Semantic distractors were the same as trained items except for the final silent symbol (e.g., 
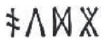
 rather than 
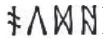
).

Performance on the recognition-memory test task is shown in Figure 5. Participants’ recognition of visually presented trained items was assessed in two analyses using *d*′ calculated with phonological distractors (Fig. 5a) and with semantic distractors (Fig. 5b). The statistical models included group and semantic marker as fixed factors and participant as a random factor. Results showed good discrimination against phonological distractors for both groups, and no significant effects were observed. However, the participant groups differed markedly with regard to discrimination of trained items against semantic distractors. The discovery-learning group showed relatively poor performance on this task, indicating that many participants in this group may have learned to ignore the final symbol during training. In contrast, the explicit-instruction group showed strong discrimination performance but only for the language in which the semantic marker conveyed systematic information (Semantic Marker × Group interaction, β = 2.67, *SE* = 0.24, *Z* = −11.19, *p* < .001). This result suggests that participants in the explicit-instruction group retained the identity of the final symbol only in conditions in which this symbol was informative.

## General Discussion

**Fig. 6.**
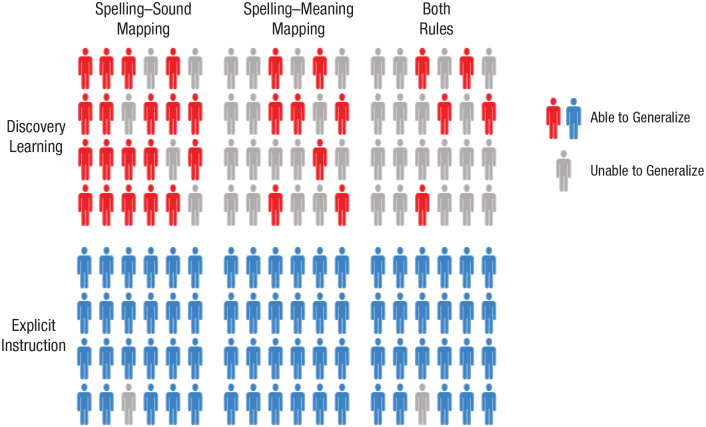
Illustration of generalization performance as a function of participant group (discovery learning [red] vs. explicit instruction [blue]) for a typical primary-school class. The criterion for being able to generalize was achieving 75% in the nonword-reading-aloud task (spelling–sound mapping) or the semantic-generalization task (spelling–meaning mapping).

Our work reveals dramatic differences in learning outcomes between participants who receive explicit instruction on the underlying regularities of a writing system and those who discover these regularities through text experience alone. Participants learned to read novel words printed in artificial alphabets over 10 days (Fig. 2). We probed participants’ retention of these trained novel words (Figs. 4 and 5) and used generalization tasks to assess their knowledge of the regularities of the writing systems (Fig. 3). The critical finding was that virtually all participants in the explicit-instruction group reached a good standard of performance on both generalization tasks, but only 5 of 24 participants in the discovery-learning group showed the same level of performance, despite having up to 18 hr of training (see Fig. 6).^1^

In contrast to generalization, instruction did not exert a strong influence on learning of individual words. Participants in both groups learned novel words to a high degree of accuracy over the 10 days of training (Figs. 2, 4, and 5), although participants reached a good standard of performance on the reading-aloud task more quickly in the presence of instruction. Further, although the discovery-learning group showed numerically lower performance on some test tasks probing item learning, this difference was significant only in the reading-aloud task (Fig. 4a). This is likely because the reading-aloud task can be performed through spelling–sound knowledge; it does not require item-specific knowledge (see also Fig. 2). These results suggest that even when good item-based learning is achieved, text experience alone does not guarantee discovery of regularities required for generalization. It is also important to note that the discovery of regularities should have been far easier in our experiment than in a naturalistic setting. Our writing systems were characterized by one-to-one mappings, a uniform structure across all exemplars, and an absence of inconsistent exemplars—features rarely shared with real writing systems. Further, our participants received multiple exemplars illustrating the underlying regularities in a structured manner, but learners outside of the laboratory encounter words relevant to particular patterns sporadically and within highly skewed frequency distributions (Yang, 2016).

Our results raise questions about the extent to which statistical learning can provide a neurocognitive basis for reading acquisition (e.g., Sawi & Rueckl, 2019). We have previously demonstrated discovery and generalization of simple underlying regularities in laboratory training studies in the absence of instruction (Taylor et al., 2017). However, the present results expose the limitations of discovery processes when writing systems increase in complexity (in this case, involving both spelling–sound and spelling–meaning regularities; see also Yim, Dennis, & Sloutsky, 2021). Indeed, emerging evidence suggests that although children do display knowledge of statistical regularities that are not typically taught explicitly, this knowledge develops very slowly and is weaker than might be expected (e.g., Pacton, Perruchet, Fayol, & Cleeremans, 2001; Treiman & Kessler, 2019). These findings suggest that discovery learning may be a relatively inefficient way of learning underlying regularities even given years of text experience.

Participants in our explicit-instruction group received approximately 30 min of training on the underlying structure of the writing systems (or ~3% of their total training time). Yet this brief session had a transformative impact on their ability to generalize 10 days later. The mechanisms that underpin the benefits of instruction are poorly understood. One influential theory suggests that instruction assists individuals to analyze and encode a stimulus optimally and to avoid false trails in a complex problem-solving space (Sweller, 2003). According to this view, instruction on its own is unlikely to be sufficient for participants to learn a new writing system; individuals will still require text experience to develop long-term stored knowledge of underlying regularities. However, instruction may act as a form of scaffold that allows learners to make the most of their text experience. Precisely how instruction interacts with text experience to create long-term knowledge structures is an important matter for future research.

These questions connect to a related debate concerning the role of explicit instruction in acquiring scientific concepts.^2^ One influential study revealed that instruction yields a substantial immediate benefit over discovery learning on children’s knowledge about the control of variables in experiments (Klahr & Nigam, 2004). Critically, this knowledge generalized to a subsequent task in which children made rich, wide-ranging judgments about science-fair posters (Klahr & Nigam, 2004). Yet other research in this domain has suggested that the benefits of instruction may be short lived (Dean & Kuhn, 2007) and that the process of discovery learning ultimately yields more robust knowledge than arises through instruction (e.g., Kuhn, 2002). Our research cannot speak to these issues concerning much longer timescales. However, the poor performance of our discovery-learning participants even after hours of training raises questions about whether a purely discovery-based approach would be viable, given the vast body of knowledge that must be acquired in learning to read. Furthermore, school-based research increasingly suggests that a fast pace of reading instruction yields superior outcomes later (Sunde, Furnes, & Lundetrae, 2020), possibly because reading skills provide the opportunity to gain vital text experience through independent reading (van Bergen, Vasalampi, & Torppa, 2020).

The critical conclusion from this work is that the way that an individual is taught has a dramatic impact on the nature of stored knowledge acquired through text experience. Some of our discovery learners were able to learn the structure of the writing system in the absence of instruction, but most were not. Further, although our findings were based on laboratory methods using adults, they are consistent with observations about children learning to read—namely, that although some children may spontaneously discover underlying regularities through their experience with printed words (Thompson, Cottrell, & Fletcher-Flinn, 1996), the majority do not (Byrne & Fielding-Barnsley, 1989). We suggest that the safest way to ensure that all learners acquire knowledge of important underlying regularities within the writing system is to offer explicit instruction of how the visual symbols of writing relate to the sounds and meanings of spoken language.

## Supplemental Material

sj-docx-1-pss-10.1177_0956797620968790 – Supplemental material for The Dramatic Impact of Explicit Instruction on Learning to Read in a New Writing System Supplemental material, sj-docx-1-pss-10.1177_0956797620968790 for The Dramatic Impact of Explicit Instruction on Learning to Read in a New Writing System by Kathleen Rastle, Clare Lally, Matthew H. Davis and J. S. H. Taylor in Psychological Science

## Transparency

*Action Editor:* Vladimir Sloutsky

*Editor:* Patricia J. Bauer

*Author Contributions*

K. Rastle, M. H. Davis, and J. S. H. Taylor conceived and designed the study; C. Lally and J. S. H. Taylor conducted the study. K. Rastle, C. Lally, M. H. Davis, and J. S. H. Taylor conceptualized the analysis; C. Lally conducted the analysis; and C. Lally and J. S. H. Taylor prepared data and documentation for OSF. K. Rastle drafted the manuscript, and all of the authors contributed critical revisions. All of the authors approved the final version of the manuscript for submission.

**Open Practices:** All data, analysis scripts, and stimuli have been made publicly available via OSF and can be accessed at https://osf.io/rtx5j/. The design and analysis plans for the experiment were not preregistered. This article has received the badges for Open Data and Open Materials. More information about the Open Practices badges can be found at http://www.psychologicalscience.org/publications/badges.

## Notes

1. One reviewer noted that instruction yielded a larger benefit for generalization of the spelling–meaning regularity than the spelling–sound regularity and wondered whether instruction may be less useful when the nature of the regularity is familiar. Our results cannot speak to this issue, though we note that both forms of regularity would be unfamiliar to children learning to read.

2. The term “direct instruction” is typically used in this literature. We have avoided this term because it can sometimes refer to a specific intervention for foundational literacy and mathematics skills.
