# Supplementary material for: The Dramatic Impact of Explicit Instruction on Learning to Read in a New Writing System
Source: Psychol Sci. 2021 Feb 26;32(4):471–84. doi: 10.1177/0956797620968790 (PMC13021060; doi:10.1177/0956797620968790)
Supplement: sj-docx-1-pss-10.1177_0956797620968790 – Supplemental material for The Dramatic Impact of Explicit Instruction on Learning to Read in a New Writing System [file sj-docx-1-pss-10.1177_0956797620968790.docx]

**
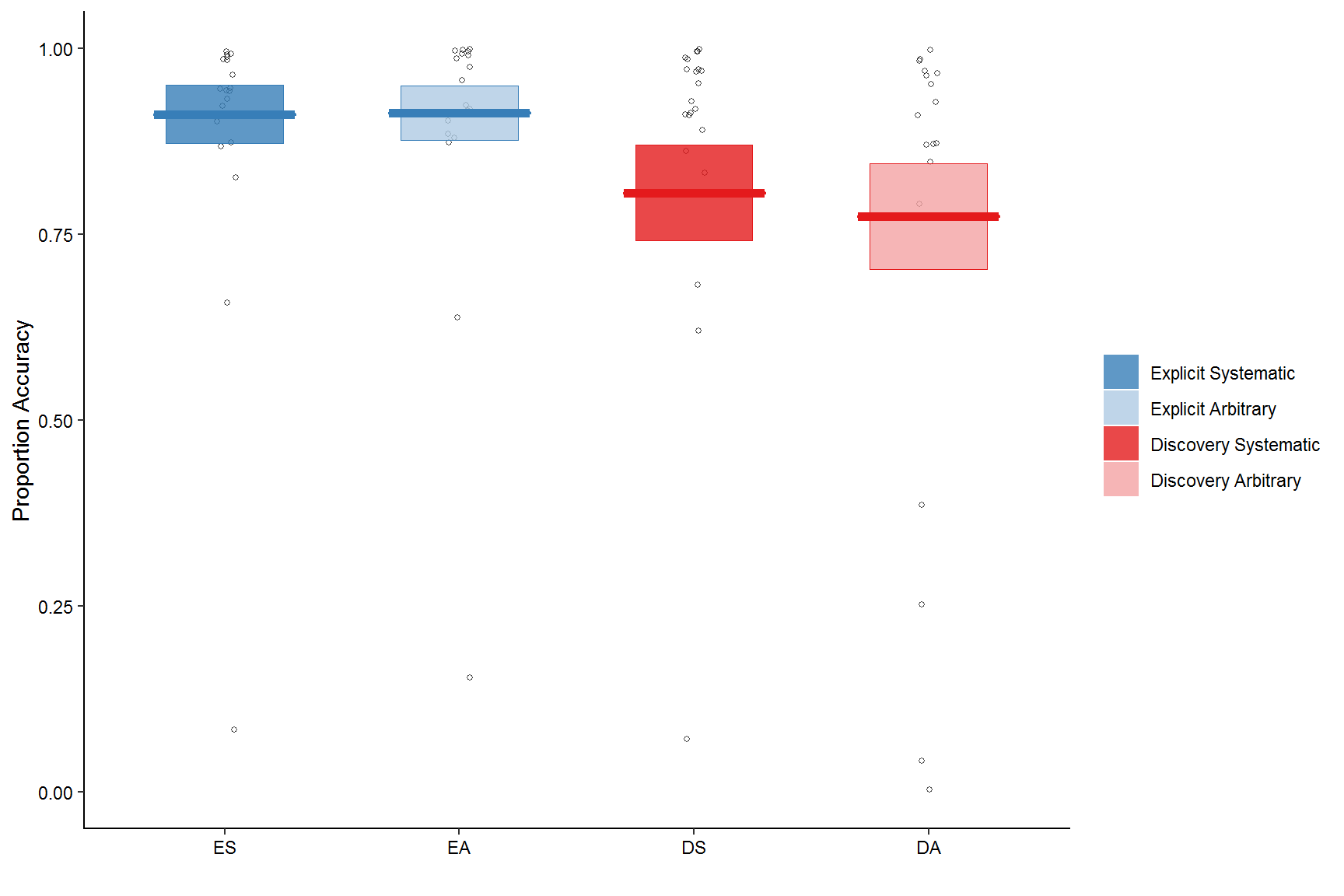
**

**Figure S1.** Performance on nonword reading aloud test task as a function of participant group and the nature of the semantic marker. Error bars display one standard error from the mean, calculated for between-subjects designs in order to compare discovery and explicit group performance.
